# Impact of implant surface modifications on long-term outcome of surgical peri-implantitis treatment: a systematic review

**DOI:** 10.3389/fdmed.2025.1661369

**Published:** 2025-09-24

**Authors:** Panagiotis Gardelis, Catherine Giannopoulou, Andreas Stavropoulos, Alkisti Zekeridou

**Affiliations:** ^1^ Division of Regenerative Dental Medicine and Periodontology, University Clinics of Dental Medicine, University of Geneva, Geneva, Switzerland; ^2^ Department of Periodontology, Faculty of Odontology, Malmo University, Malmo, Sweden; ^3^ Department of Periodontology, Blekinge Hospital, Karlskrona, Sweden; ^4^ Division of Conservative Dentistry and Periodontology, University Clinic of Dentistry, Medical University of Vienna, Vienna, Austria; ^5^ Department of Periodontology, School of Dental Medicine, University of Bern, Bern, Switzerland

**Keywords:** peri-Implantitis, surgical peri-implantitis treatment, treatment outcome, explantation, implant surface, long-term outcomes, bone loss, implant survival

## Abstract

**Introduction:**

Peri-implantitis is an inflammatory disease that compromises peri-implant tissues and supporting bone, potentially leading to implant loss. Although several surgical treatment strategies have been proposed, it remains unclear whether implant surface characteristics (smooth vs. rough) influence long-term treatment outcomes.

**Methods:**

A systematic review was conducted to evaluate clinical studies with a minimum follow-up of 3 years that assessed the outcomes of surgical treatment of peri-implantitis in relation to implant surface type. Data extraction focused on recurrence of peri-implantitis, implant survival, clinical parameters, radiographic outcomes, and the type of surgical approach used (reconstructive vs. non-reconstructive).

**Results:**

Seventeen clinical studies were included. Outcomes varied according to implant surface characteristics. Rough (modified) surfaces were generally associated with a higher risk of recurrence of peri-implantitis and implant loss compared with smooth (machined/turned) surfaces. Reconstructive surgical approaches, especially those involving bone grafts and membranes, demonstrated more favorable outcomes compared with non-reconstructive approaches.

**Discussion:**

Despite observed trends, the certainty of the evidence remains low due to heterogeneity between studies, small sample sizes, and methodological limitations. Further well-designed long-term clinical trials are needed to clarify the role of implant surface characteristics in the long-term success of peri-implantitis surgical treatment.

**Systematic Review Registration:**

PROSPERO (CRD420251129791).

## 1 Introduction

Dental implants have significantly advanced oral rehabilitation, providing highly predictable solutions for tooth replacement. For instance, a recent systematic review reported that long-term prospective studies on dental implants show high survival rates, typically exceeding 90% over 5–10 years and remaining around 78% after imputation at 20 years follow-up. In addition, five retrospective studies with ≥20 years of follow-up reported an implant survival rate of approximately 88%, including multifactorial causes (1).

However, despite the high survival rates, biological complications at implants are rather common. In particular, peri-implantitis, which is characterized by peri-implant mucosal inflammation and progressive bone loss, affects approximately 19.53% of patients and 12.53% of implants, highlighting its relevance in clinical practice (2). As the main etiological factor for peri-implantitis is the oral biofilm, microbial to implant surface interactions seem to play an important role in disease pathogenesis. Indeed, surface modifications (e.g., sandblasting, acid-etching, anodization, etc) aiming in enhancing implant surface bioactivity, substantially impact on microbial colonization and biofilm development (3–7). Indeed, although the incidence of peri-implantitis seems not to differ between modified and non-modified (i.e., turned) implants in the clinic, progression and severity of peri-implantitis appear linked to implant surface properties; specifically, pre-clinical *in vivo* studies indicate a faster disease progression at modified implants compared with turned implants, as well as differences in disease progression among various modified surfaces (5, 7). Moreover it seems that implant surface characteristics may impact on treatment outcomes both in the short-term but also on the long-term, with implants with a modified surface demonstrating less positive results and higher recurrence rates (3, 8, 9).

Despite technological advancements and improved treatment approaches, the impact of implant surface modifications on peri-implantitis outcomes remains unclear. Therefore, this systematic review aims to evaluate whether varying implant surface topographies influence clinical and radiographic outcomes following surgical peri-implantitis treatment in humans. The findings may offer critical insights guiding the selection of implant surface characteristics to enhance treatment efficacy.

## 2 Materials and methods

### 2.1 Study design

This review was performed following the Preferred Reporting Items for Systematic Reviews and Meta-Analyses (PRISMA) guidelines and was registered in PROSPERO (ID: CRD420251129791).

### 2.2 Search strategy

To identify relevant studies, we systematically searched PubMed, Embase, and the Cochrane Library. The search strategy was carried out in English language from database inception for articles published between 2000 and 2025. Two investigators (AZ and PG) independently reviewed the search results and screened the titles and abstracts. Full texts of all potentially eligible studies were obtained. In PubMed, the following search strategy was used: “(Periimplantitis OR peri-implantitis OR peri implantitis OR periimplant OR peri-implant OR peri implant) AND (treatment outcome OR therapy OR surgical treatment OR regenerative OR regeneration OR tissue regeneration OR reconstructive surgery OR bone graft OR bone substitute OR membranes OR surgical flap OR open flap debridement OR resective OR implantoplasty OR surface decontamination) AND (surface characteristics OR surface roughness OR material characteristics OR titanium surface OR implant types OR implant surfaces OR surface topography OR surface analysis) AND (implant survival OR bone loss OR recurrence OR retreatment OR radiographic stability OR long-term OR 3 years OR follow-up).” This search strategy was adapted to suit the other electronic sources. The reference lists of retrieved articles were also checked to identify additional studies of interest. Any inconsistencies were resolved by consensus with a third investigator (CG). The complete search strategies for all databases are provided in Appendix 2.

### 2.3 Criteria for considering studies for this review

#### 2.3.1 Study design

Randomized controlled trials, prospective studies, retrospective studies, case-control studies, and case series were included. No specific cut-off criteria for sample size were applied, given the limited availability of data. Additionally, two case series with very small sample sizes were included due to their clinical relevance. Eligibility required that included studies explicitly reported the implant surface type(s) of the implants investigated.

#### 2.3.2 Population

Human studies. Patients with osseointegrated dental implants diagnosed with peri-implantitis, treated surgically, with a follow-up period of at least 3 years (or an average ≥3 years).

#### 2.3.3 Intervention

Surgical therapy for peri-implantitis.

#### 2.3.4 Comparator

Different implant surface types, characterized by variations in macro-, micro-, and nano-scale surface roughness, topography, and material composition. Surfaces were categorized as non-modified (i.e turned, smooth, machihed), modified (rough), or mixed (hybrid), depending on their reported surface characteristics.

### 2.4 Outcomes

#### 2.4.1 Primary outcome

• Percentage of implants with recurrence of peri-implantitis requiring re-treatment or explantation or simply defined as treatment failure by the authors.

### 2.4.2 Secondary outcomes

• Implant loss (due to any reason)
• Disease resolution defined by reduction of probing depth (PD) without bleeding on probing (BOP) or suppuration
• Radiographic bone loss or gain assessed by mean changes in bone levels or percentage of implants with stable bone levels post-treatment
• Mean probing depth (PD) post-treatment

Subgroup synthesis: The outcomes were further stratified based on implant surface types and surgical approach:

1. Turned (machined/non-modified surfaces)
  • a. Non-reconstructive surgical approach
  • b. Reconstructive surgical approach (regardless the technique or materials used)
2. Modified (rough surfaces)
  • a. Non-reconstructive surgical approach
  • b. Reconstructive surgical approach (regardless the technique or materials used)
3. Mixed or unspecified surfaces
  • a. Non-reconstructive surgical approach
  • b. Reconstructive surgical approach (regardless the technique or materials used)

### 2.5 Data collection

Two investigators independently extracted key data from the included articles. The inter-rater agreement for study selection was assessed using Cohen's kappa statistics. Inter-rater reliability was assessed using Cohen's kappa statistic on a subset of 20% of studies, yielding a kappa of 0.85, indicating a high level of agreement. Discrepancies were resolved through discussion or consultation with a third reviewer (CG). For each article, we extracted study features (i.e., study design, year of publication, number of enrolled patients), type of intervention, and outcome measures. Correct data extraction was controlled in a subset of randomly selected studies by the third investigator.

### 2.6 Assessment of risk of bias

Two investigators independently appraised the risk of bias of the included studies using the Cochrane Risk of Bias Tool 2.0 (RoB2) for RCTs. For non-RCTs the Risk of Bias in Non-randomized Studies of Interventions (ROBINS-I) tool was used. Any inconsistencies were resolved by consensus with a third investigator (CG).

### 2.7 Data synthesis

Preliminary analyses of available data revealed high heterogeneity, precluding meaningful meta-analysis. Therefore, a narrative synthesis was conducted. These limitations included significant heterogeneity in implant surface types, surgical techniques, and reported outcome measures across studies. To enhance clarity and readability, findings were systematically summarized in tables according to pre-defined outcomes and subgroup analyses.

Data extraction was performed separately for each treatment group within studies containing multiple groups, while data from studies with a single treatment group were extracted accordingly. Results were categorized based on implant surface types and surgical approaches as follows:

1. Turned (machined/non-modified surfaces) a. Non-reconstructive surgical approach b. Reconstructive surgical approach (regardless the technique or materials used)
2. Modified (rough surfaces) a. Non-reconstructive surgical approach b. Reconstructive surgical approach (regardless the technique or materials used)
3. Mixed or unspecified surfaces a. Non-reconstructive surgical approach b. Reconstructive surgical approach (regardless the technique or materials used)

Findings were systematically summarized in tables according to pre-defined outcomes and subgroup analyses to enhance clarity and readability.

Within each treatment group, data were systematically collected on key parameters, including sample size (number of participants and implants), criteria used to define peri-implantitis, type of bone substitute, membrane used (if applicable), follow-up periods, implant system, and implant surface characteristics**.**

## 3 Results

### 3.1 Study selection

**Figure 1:**
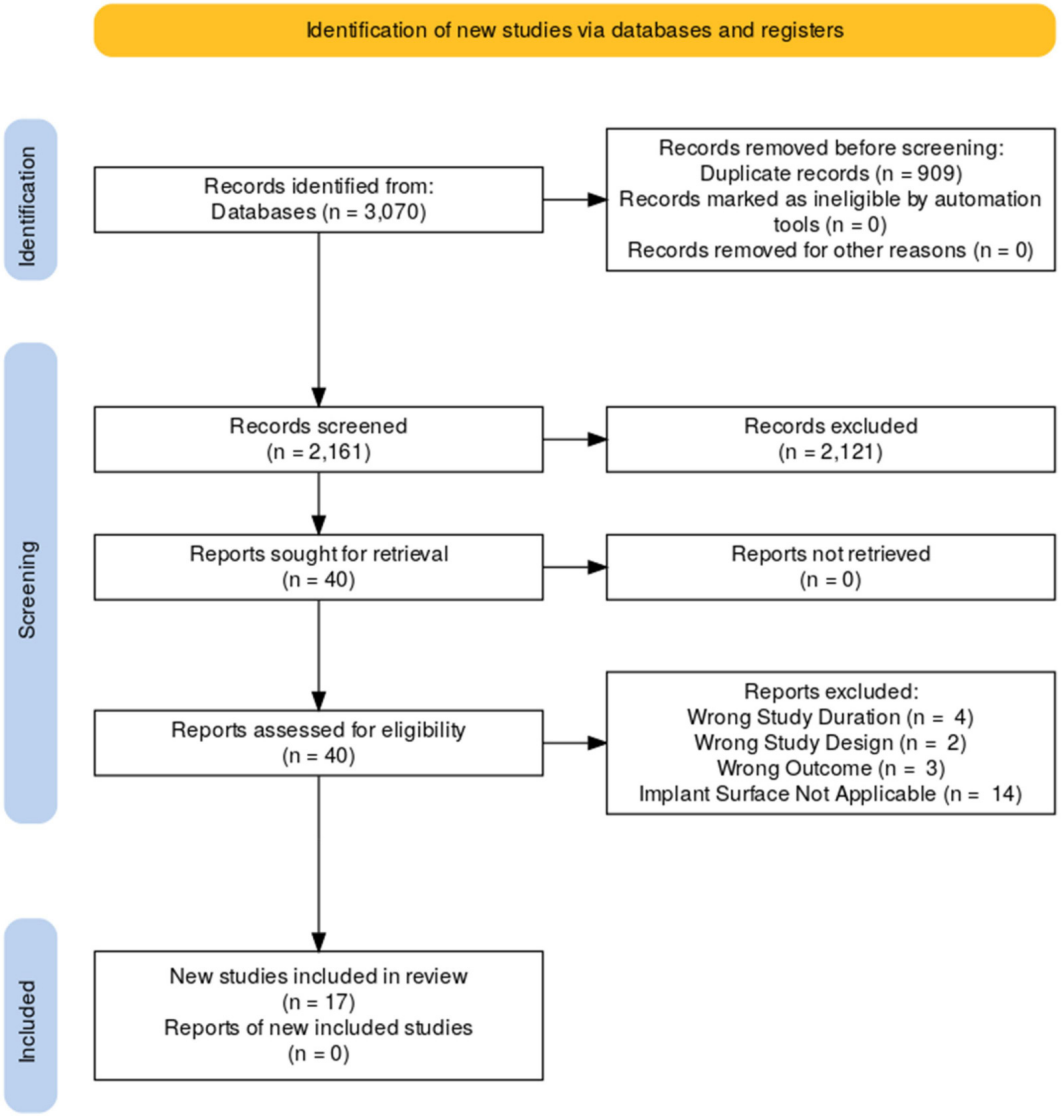
PRISMA flow diagram.

The literature search process is illustrated in the flowchart below (Figure 1). In total, there are 17 studies included in the analysis (8–24). Among them, 8 are prospective cohort studies, 3 are retrospective cohort studies, 1 are randomized controlled trials. The remaining studies include 1 each of the following types: prospective clinical study, retrospective observational study, and prospective case series. A detailed description of the study characteristics can be found in the results in Tables 1a,b.

**Table 1a:** Summary of clinical studies evaluating treatments of peri-implantitis: surface types, materials, and outcomes.

Author (year)	Implant surface	Implant brand	Patients (initial/follow-up)	Implants (initial/follow-up)	Type of treatment	Materials used	Follow-up period	Surface impact
Aghazadeh et al. (2022) (32)	• Mixed (Turned and medium-rough)	• Not explicitly stated, categorized by surface type	• 45 (AB:22/16, BDX:23/23)	• 75 (AB:36/25, BDX:39/38)	• Reconstructive surgery (autogenous bone, xenograft, collagen membrane)	• Autogenous bone, xenograft (Bio-Oss), collagen membrane (OsseoGuard), titanium curettes, hydrogen peroxide, azithromycin, CHX rinse	• 5 years	• No significant difference (turned vs. medium-rough surfaces)
Carcuac et al. (2020) (15)	• Mixed (modified/non-modified)	• Not specified, classified as non-modified/modified	• 100/73	• 179/130	• Open-flap debridement, surface decontamination, pocket elimination	• Systemic antibiotics, local antiseptics	• 5 years	• Modified surfaces higher recurrence risk (OR 5.1, 95% CI: 1.6–16.5)
Deppe et al. (2007) (18)	• Mixed (rough predominantly)	• IMZ, Frialit-2, Brånemark, Straumann screw-type	• 32	• 73 (Conventional:34, Laser:39)	• Conventional vs. CO₂ laser-assisted (soft tissue resection/augmentation)	• CO₂ laser, air-powder abrasive, *β*-TCP/autogenous bone, Gore-Tex membrane	• 5 years	• Superior outcomes with CO₂ laser in non-reconstructive therapy
Jemt & Eriksson (2021) (19)	• Mixed (Turned and moderately rough surfaces)	• Brånemark (turned), TiUnite, Astra Tech OsseoSpeed, Lifecore RBM	• 122 (initially 134, 12 lost)	• 614/453	• Non-reconstructive surgery (mechanical cleaning, osseous recontouring, antibiotics)	• Mechanical debridement, hydrogen peroxide 10%, systemic antibiotics	• Mean 7.3 years	• No significant difference (turned vs. moderately rough surfaces)
Khoury & Buchmann (2001) (20)	• Rough surfaces	• IMZ, Frialit-2 (Friadent GmbH)	• 25	• 41	• Reconstructive surgery (autogenous bone ± membranes)	• Autogenous bone, ePTFE/Bioabsorbable barriers, CHX, citric acid, hydrogen peroxide, systemic antibiotics	• 3 years	• No surface comparison (all rough)
La Monaca et al. (2024) (22)	• Rough (TiUnite surface)	• Nobel Biocare (Brånemark System, Göteborg)	• 34/23	• 34/20	• Reconstructive surgery (MDBA, resorbable membrane, chemical/mechanical decontamination)	• MDBA (Puros), collagen membrane (Bio-Gide), hydrogen peroxide, CHX solution, tetracycline hydrochloride, systemic antibiotics (Amox/clav, Metronidazole)	• 10 years	• All rough (TiUnite); no surface-specific comparison
La Monaca et al. (2018) (21)	• Rough (TiUnite surface)	• Nobel Biocare (Brånemark System, Göteborg)	• 34	• 34	• Reconstructive surgical therapy (MDBA, resorbable collagen membrane, chemical/mechanical decontamination)	• MDBA (Puros), resorbable membrane (Bio-Gide), hydrogen peroxide, CHX 0.2%, tetracycline hydrochloride, systemic antibiotics	• 5 years	• All rough (TiUnite); no surface comparison
Leonhardt et al. (2003) (23)	• Turned surfaces	• Brånemark System (Nobel Biocare)	• 9	• 26	• Surgical non-reconstructive therapy + systemic antibiotics	• Hydrogen peroxide 10%, individualized antibiotics (metronidazole, amoxicillin, tetracycline, ciprofloxacin, clindamycin), CHX rinse	• 5 years	• All turned surfaces; not directly analyzed
Mercado et al. (2018) (24)	• Rough (Micro-rough)	• Branemark TiUnite (46.66%), Astra Tech (26.66%), Straumann (10%), Others (16.66%)	• 30	• 30	• Regenerative surgery (DBBMC, EMD, doxycycline, EDTA, ultrasonic scaler)	• DBBMC, EMD, doxycycline, EDTA 24%, ultrasonic scaler, chlorhexidine 0.12%	• 36 months	• Not specifically analyzed (micro-rough surfaces)
Noelken et al. (2023) (25)	• Mixed (rough predominantly)	• Straumann, Ankylos, Brånemark, NobelActive, NobelPerfect, Frialit I, OsseoSpeed, Camlog, ICX	• 18	• 24	• LAPIDER (laser-assisted regeneration, autogenous bone, CT graft)	• Er:YAG laser, autogenous bone chips, doxycycline, CT graft, resorbable sutures	• 3 years	• No direct comparison; rough surfaces only
Roccuzzo et al. (2017) (26)	• Rough (SLA and TPS)	• Straumann (SLA, TPS)	• 26/24	• 26/24	• Regenerative surgery (DBBMC, EDTA, CHX)	• DBBMC (Bio-Oss Collagen), EDTA 24%, CHX gel 1%, antibiotics (Amoxicillin/clavulanic acid)	• 7 years	• SLA better clinical outcomes than TPS (significant)
Roccuzzo et al. (2020) (27)	• Rough (SLA and TPS)	• Straumann (SLA, TPS)	• 26/14	• 26/14	• Regenerative surgery (DBBMC, EDTA, CHX)	• DBBMC (Bio-Oss Collagen), EDTA 24%, CHX gel 1%, antibiotics (Amoxicillin/clavulanic acid)	• 10 years	• SLA superior survival/outcomes compared to TPS
Roccuzzo et al. (2021) (28)	• Rough (SLA)	• Straumann (SLA implants)	• 75/51	• 75/64	• Reconstructive surgery (DBBMC, EDTA, CHX gel, titanium curettes, connective tissue graft)	• DBBMC, EDTA 24%, CHX 1% gel, titanium curettes, titanium brush, connective tissue graft, systemic antibiotics	• 5 years	• Uniform (all SLA surfaces)
Romandini et al. (2024) (16)	• Mixed (Turned and Modified)	• Nobel Biocare (74.2%), Astra Tech (18%), Straumann (6.7%), Neoss (1.1%)	• 149	• 267	• Non-reconstructive surgical therapy (titanium-coated curettes, systemic antibiotics)	• Titanium-coated curettes, saline/CHX gauze, selective systemic antibiotics (Amoxicillin)	• Mean 7 years (range 1-18 years)	• Modified surfaces significantly higher implant loss risk (HR = 4.5)
Roos-Jansåker et al. (2011) (29)	• Primarily machined, few modified (rough)	• Brånemark (majority), Astra Tech (minority)	• 38/32	• 65/56	• Reconstructive surgery (Algipore ± membrane)	• Algipore, Osseoquest membrane, H₂O₂ 3%, antibiotics (amoxicillin, metronidazole), CHX 0.1% rinse	• 3 years	• No significant impact reported (mostly machined)
Roos-Jansåker et al. (2014) (30)	• Primarily machined, few modified (rough)	• Brånemark (majority), Astra Tech (minority)	• 38/25	• Not initially specified/45	• Reconstructive surgery (Algipore ± membrane)	• Algipore, Osseoquest membrane, H₂O₂ 3%, antibiotics (amoxicillin, metronidazole), CHX rinse	• 5 years	• No significant impact (mostly machined surfaces)
Schwarz et al. (2009) (31)	• Mixed (machined and rough surfaces)	• Brånemark, Camlog, ITI, KSI Bauer Schraube, Zimmer, ZL-Duraplant	• 22/19	• 22/19	• Regenerative surgery (NBM + CM vs. NHA)	• NBM (BioOss), CM (BioGide), NHA (Ostim), plastic curettes, CHX 0.2%	• 48 months	• Surface-specific outcomes not detailed

**Table 1b:** Comparative outcomes of clinical studies on peri-implantitis treatments: implant surfaces, secondary outcomes, and long-term surface impact.

Author (year)	Implant loss (%)	Disease resolution	Radiographic bone loss/gain	Mean PD post-treatment	Subgroup analysis (by surface & approach)	Conclusions surface impact
Aghazadeh et al. (2022) (32)	• AB: 1 fractured, BDX: 1 fractured (both initially)	• PD reduction AB: 1.7 mm, BDX: 2.8 mm at 5 years	• AB: −0.7 mm (loss), BDX: + 1.6 mm (gain)	• AB: PD reduced by 1.7 mm, BDX: by 2.8 mm at 5 years	• BDX superior to AB; reconstructive approach only, no surface-specific outcomes detailed	• BDX superior to AB; supportive maintenance every 3 months emphasized. No significant difference (turned vs. medium-rough surfaces)
Carcuac et al. (2020) (15)	• 20.8%	• Not explicitly combined; PD ≥ 6 mm at 1 year = increased recurrence (OR 7.4)	• Stable from year 1; further loss >1 mm in 13.1%	• 5.0 mm (stable from year 1 at 4.9 mm)	• Non-modified: 17% recurrence, Modified: 52% recurrence; no explicit surgical subgroup	• Surgical treatment effective, but 44% recurrence; modified surfaces, deep residual PD major risk factors. Modified surfaces higher recurrence risk (OR 5.1, 95% CI: 1.6–16.5)
Deppe et al. (2007) (18)	• 17.8%	• Superior PD reduction laser (1.0–2.4 mm at 4 months)	• Laser better short-term bone gain; long-term similar outcomes	• Laser: 1.0–2.4 mm, Conventional: 2.4–7.9 mm (long-term)	• Laser superior in soft tissue resection; similar in augmentation	• Laser beneficial in non-reconstructive procedures; similar long-term results in augmentation. Superior outcomes with CO₂ laser in non-reconstructive therapy
Jemt & Eriksson (2021) (19)	• 16.7%	• Not explicitly reported; primarily bone-level outcomes	• Increased bone loss post-treatment (0.26 mm/year)	• PD not explicitly reported	• No significant differences between surface types; increased bone loss post-treatment	• Surgical treatment ineffective long-term; bone loss accelerated post-treatment, edentulous worse prognosis. No significant difference (turned vs. moderately rough surfaces)
Khoury & Buchmann (2001) (20)	• 0% explicitly reported	• PD improvement: Bone alone 8.0→2.9 mm, worse with membranes (7.7→5.1 mm)	• Significant bone gain (bone alone 3.2 mm, membranes lower)	• Bone alone: 2.9 mm; Non-resorbable: 2.8 mm; Bioabsorbable: 5.1 mm	• Bone alone superior; membranes caused high complications	• Bone grafting alone effective; membranes did not enhance outcomes, increased complications. No surface comparison (all rough)
La Monaca et al. (2024) (22)	• 8.8%	• Composite success: 53%, PD significantly reduced to 2.95 mm at 10 years	• Mean bone gain: 1.07 mm (stable 10 years)	• Reduced from 6.33 mm to 2.95 mm at 10 years	• Reconstructive approach only; stable bone gain, PD improvement, composite success 53%	• Long-term reconstructive therapy stable; supportive therapy crucial, defect severity not significant. All rough (TiUnite); no surface-specific comparison
La Monaca et al. (2018) (21)	• 0%	• PD reduced initially, increased by 5 years (4.62 mm, nonsignificant from baseline)	• Initial significant bone gain (1 year), gradual loss by 5 years	• 4.62 mm at 5 years (from 5.93 mm baseline)	• Reconstructive surgery initially effective, unstable outcomes long-term	• Initial benefits lost over time, no implants lost; unpredictable outcomes long-term. All rough (TiUnite); no surface comparison
Leonhardt et al. (2003) (23)	• 27%	• Significant reduction plaque (100%→11%) and bleeding (100%→5%)	• Stable in 9 implants, gain in 6 implants, loss in 4 implants	• Not explicitly reported; clinical signs significantly improved	• Non-reconstructive approach; success 58%, significant clinical improvements	• Limited success (58%); smoking negatively impacted outcomes. All rough surfaces; not directly analyzed
Mercado et al. (2018) (24)	• 0%	• Mean PD from 8.9 mm to 3.5 mm; BOP/suppuration from 100% to 20%	• Bone gain (6.92 mm to 2.60 mm mean bone loss at 36 months)	• Reduced from 8.9 mm to 3.5 mm at 36 months	• Regenerative (reconstructive) approach only, significant PD reduction and bone gain; success 56.7%	• Regenerative treatment effective (56.7% success); regular supportive therapy critical. Not specifically analyzed (micro-rough surfaces)
Noelken et al. (2023) (25)	• 8.3%	• PD significantly reduced (5.05 to 3.08 mm), BOP 100% to 36.4%	• Significant bone gain (Interproximal 3.1 mm, Buccal 3.5 mm, Lingual 1.46 mm)	• Final PD 3.08 mm	• LAPIDER effective for severe defects; significant hard/soft tissue regeneration	• LAPIDER provided substantial regeneration and aesthetic improvements. No direct comparison; rough surfaces only
Roccuzzo et al. (2017) (26)	• 16.7% overall (SLA:16.7%, TPS:28.6%)	• Significant PD reduction, SLA (3.2 mm), TPS (3.4 mm); BOP SLA (7.5%), TPS (30%)	• Significant bone fill SLA (2.1 mm gain), TPS (2.0 mm gain)	• SLA: 3.2 mm, TPS: 3.4 mm	• Regenerative approach better for SLA vs. TPS implants	• DBBMC effective; SLA surfaces significantly better clinical outcomes than TPS.
Roccuzzo et al. (2020) (27)	• SLA:20%, TPS:45%	• Significant PD reduction SLA (3.2 mm), TPS (3.4 mm); BOP significantly reduced	• SLA substantial gain (2.7 mm), TPS moderate gain (2.0 mm)	• SLA: 3.2 mm, TPS: 3.5 mm	• SLA implants superior long-term survival/outcomes	• DBBMC stable outcomes; SLA significantly better than TPS long-term
Roccuzzo et al. (2021) (28)	• 17%	• PD 6.89 mm to 4.06 mm; BOP 70.6% to 17.2%	• Substantial bone fill; no numeric specifics reported	• Reduced from 6.89 mm to 4.06 mm at 5 years	• Reconstructive approach only; SLA implants only, survival 80%, success 45.3%, significant PD reduction	• Reconstructive protocol effective; adherence to supportive therapy significantly improves outcomes.
Romandini et al. (2024) (16)	• 19.9%	• Not explicitly; high recurrence, retreatment common (24.3%)	• Mean additional bone loss: 0.97 mm; >1 mm loss in 42.4%	• Not explicitly detailed post-treatment; baseline deepest PD 7.8 mm	• Turned surfaces significantly better prognosis; modified surfaces high loss risk (HR = 4.5)	• High recurrence; surface type crucial predictor, severe baseline bone loss/suppuration increase loss risk. Modified surfaces significantly higher implant loss risk (HR = 4.5)
Roos-Jansåker et al. (2011) (29)	• 0%	• Clinical measures not explicitly detailed	• Stable bone fill: bone graft (1.3 mm), membrane (1.6 mm), no significant difference	• Not explicitly reported	• Stable outcomes, no significant membrane advantage	• Algipore ± membrane effective, stable bone fill, membrane no additional advantage. No significant impact reported (mostly machined)
Roos-Jansåker et al. (2014) (30)	• 0%	• PD reduction: 3.0–3.3 mm, BOP significantly reduced	• Bone gain stable: 1.1–1.3 mm, no significant membrane advantage	• 2.6–2.7 mm	• No significant advantage with membrane	• Stable results; membrane no additional advantage over bone substitute alone. No significant impact (mostly machined surfaces).
Schwarz et al. (2009) (31)	• 0% explicitly reported (1 discontinued)	• NBM + CM: PD reduction 2.5 mm, NHA: 1.1 mm, BOP significantly reduced	• NBM + CM superior bone fill compared to NHA	• NBM + CM: 4.6 mm, NHA: 5.8 mm	• NBM + CM significantly better than NHA	• NBM + CM superior long-term clinical/radiographic outcomes vs. NHA. Surface-specific outcomes not detailed.

AB, autogenous bone; BDX, bovine-derived xenograft; BOP, bleeding on probing; CAL, clinical attachment level; CHX, chlorhexidine; CI, confidence interval; CM, collagen membrane; CO₂, carbon dioxide; CT, connective tissue; DBBMC, deproteinized bovine bone mineral with collagen; DH, defect height; EMD, enamel matrix derivative; ePTFE, expanded polytetrafluoroethylene; H₂O₂, hydrogen peroxide; HR, hazard ratio; LAPIDER, laser-assisted peri-implant defect regeneration; MBL, marginal bone level; MDBA, mineralized dehydrated bone allograft; NBM, natural bone mineral; NHA, nanocrystalline hydroxyapatite; OR, odds ratio; PD, probing depth; P-IS, peri-implantitis surgery; SLA, sandblasted large grit acid-etched; SOP, suppuration on probing; TPS, titanium plasma-sprayed; β-TCP, beta-tricalcium phosphate.

### 3.2 Study populations

#### 3.2.1 Peri-implantitis

Across the 17 studies analyzed, various diagnostic criteria have been employed to identify peri-implantitis, reflecting differences in study designs and clinical considerations (8–24). The most commonly reported diagnostic parameters include probing depth (PD), bleeding on probing (BOP), suppuration, and radiographic evidence of bone loss.

#### 3.2.2 Probing depth (PD)

A probing depth threshold of ≥6 mm is frequently used as a criterion to identify peri-implantitis, as observed in studies by Carcuac et al., Romandini et al., and Roccuzzo et al. (15, 16, 26–28). Other studies, such as Aghazadeh et al. and Noelken et al., set a threshold of ≥5 mm, which is similar to the >4 mm threshold considered indicative of disease by Mercado et al. and Schwarz et al. (24, 25, 31, 32). This variation highlights differences at diagnosis across studies.

#### 3.2.3 Bleeding on probing (BOP) and suppuration

The presence of BOP and/or suppuration was consistently reported as a diagnostic marker in all studies. It serves as an indicator of ongoing inflammation and peri-implant tissue destruction. Studies such as La Monaca et al. and Khoury & Buchmann emphasize the importance of these clinical signs in combination with radiographic findings for accurate diagnosis (20–22).

#### 3.2.4 Radiographic bone loss

Radiographic evaluation of bone loss is another widely accepted criterion for peri-implantitis diagnosis. The threshold for bone loss varies among studies, with the most commonly reported value being ≥3 mm, as seen in Romandini et al. (16). Other studies, including Carcuac et al. and Aghazadeh et al., defined progressive bone loss based on post-treatment changes or specific defect characteristics, such as angular defects of ≥3 mm (15, 32). A more conservative threshold of ≥1.8 mm was applied in studies such as Roos-Jansåker et al., reflecting the variability in bone loss progression (29, 30).

#### 3.2.5 Variability in diagnostic criteria

Despite a general agreement on the primary diagnostic signs—probing depth, BOP/suppuration, and radiographic bone loss—variability exists in the specific thresholds and additional criteria applied across studies.

A detailed overview of the case definitions used to include patients with peri-implantitis in each study (treatment group) is provided in Table 2.

**Table 2:** Definitions of periimplantitis.

Study	Definition of periimplantitis
Aghazadeh et al., 2022 (32)	Probing pocket depths of at least 5 mm, presence of bleeding on probing and/or suppuration, radiographic bone loss of 2 mm or more from implant placement to screening, and an angular peri-implant bone defect of 3 mm or greater
Carcuac et al., 2020 (15)	Probing pocket depths of 6 mm or more, presence of bleeding on probing, reduced marginal bone level and progressive bone loss greater than 1 mm post-treatment
Deppe et al., 2007 (18)	Probing pocket depths of at least 5 mm, presence of bleeding on probing, radiographic evidence of progressive vertical bone loss, and clinical signs of inflammation
Jemt etal., 2021 (19)	Bone loss exceeding 0.4 mm, mucosal inflammation, presence of plaque and/or suppuration, and radiographic evidence of marginal bone loss
Khoury et al., 2001 (20)	Bone loss of more than 50% of the implant length, augmented probing depths, bleeding on probing, and radiographic evidence of intrabony defects
La Monaca et al., 2018 (21)	Progressive bone loss of 3 mm or more detected on radiographs, the presence of bleeding on probing and/or suppuration, and probing depths of at least 5 mm
La Monaca et al., 2024 (22)	Progressive angular bone loss of at least 3 mm beyond crestal bone level changes, the presence of bleeding on gentle probing and/or suppuration, and implants in function for more than 12 months
Leonhardt et al., 2003 (23)	Marginal bone loss of at least three implant threads compared to baseline radiographs, bleeding on probing and/or suppuration from peri-implant sulci, and microbiological confirmation of peri-implant pathogens
Mercado et al., 2018 (24)	Probing pocket depths exceeding 4 mm, the presence of bleeding on probing and/or suppuration, a minimum radiographic bone loss of 20%, and implants that have been in function for at least 2 years
Noelken et al., 2023 (25)	Probing pocket depths greater than 5 mm, the presence of bleeding on probing and suppuration, and radiographically confirmed bone loss
Roccuzzo et al., 2017 (26)	Probing pocket depths of at least 6 mm, no implant mobility, bleeding on probing and/or suppuration, and radiographic bone loss exceeding three implant threads compared to baseline
Roccuzzo et al., 2020 (27)	Probing pocket depths of 6 mm or greater, the presence of bleeding on probing and/or suppuration, radiographic bone loss beyond crestal changes, and the absence of implant mobility
Roccuzzo et al., 2021 (28)	Probing pocket depths reach or exceed 6 mm, bleeding on probing, radiographic evidence of progressive bone loss, and the presence of pus or inflammation
Romandini et al., 2024 (16)	Probing pocket depths of 6 mm or more, bleeding and/or suppuration on probing, and radiographic evidence of marginal bone loss equal to or greater than 3 mm
Roos-Jansåker et al., 2011 (29)	Radiographic bone loss of at least 1.8 mm following the first year in function, the presence of bleeding and/or pus on probing, and inclusion criteria of non-mobile implants
Roos-Jansåker et al., 2014 (30)	Radiographic bone loss of at least 3 threads (≥1.8 mm), the presence of a vertical defect component, and bleeding on probing and/or suppuration
Schwarz et al., 2009 (31)	Probing pocket depths greater than 4 mm, presence of bleeding on probing and/or suppuration, radiographic evidence of bone loss, and an intrabony defect component of at least 3 mm

### 3.3 Primary outcome: recurrence and treatment failure

The included studies demonstrated that implant surface characteristics influenced recurrence rates following surgical peri-implantitis treatment. Modified (rough) surfaces consistently showed higher recurrence compared with turned (machined) surfaces. Carcuac et al. reported an overall recurrence of 44%, with a significantly increased risk for modified surfaces (OR 5.1) (15). Similarly, Romandini et al. found a retreatment rate of 24.3%. In contrast, studies involving turned surfaces, such as Leonhardt et al., reported more stable outcomes (23). These findings indicate that surface roughness is a key determinant of recurrence and long-term treatment stability. Studies by Schwarz et al., Mercado et al. and Noelken et al. documented relatively stable outcomes without explicitly reporting significant recurrence rates (24, 25, 31).

### 3.4 Secondary outcomes

#### 3.4.1 Implant loss

Implant loss was more frequent among rough surface implants, especially TPS, with Roccuzzo et al. reporting loss in 45% of TPS implants (27), and Leonhardt et al. reporting 27% for turned surfaces (23). SLA surfaces demonstrated better survival than TPS, with 20% vs. 45% loss after 10 years (27). Modified surfaces were identified as a strong predictor of implant loss (HR 4.5) (16). Turned surfaces generally exhibited lower long-term loss risk, around 20%, compared to modified ones (16). Lower implant loss rates were generally associated with reconstructive surgical approaches, as observed by Noelken et al. 8.3% (25) and La Monaca et al. (22), 8.8%.

#### 3.4.2 Disease resolution and probing depth (PD)

Reconstructive surgery generally improved PD irrespective of surface, but rough surfaces demonstrated greater variability. Mercado et al. (2018) reported PD reduction from 8.9 mm to 3.5 mm on micro-rough implants (24), while Noelken et al. achieved PD reduction from 5.05 mm to 3.08 mm in predominantly rough implants (25) identifying disease resolution. Roccuzzo et al. observed significant PD improvements for SLA implants compared with TPS (26, 27). Turned implants Leonhardt et al., also demonstrated significant PD reduction (23). Conversely, non-reconstructive surgical approaches such as that of Deppe et al. with predominantly rough surfaces showed initial short-term PD reductions with inconsistent long-term stability (18).

#### 3.4.3 Radiographic bone changes

Bone regeneration outcomes were surface-dependent. Reconstructive procedures around rough implants, particularly SLA, showed consistent bone gain (Roccuzzo et al. + 2.1 mm; + 2.7 mm) (26, 27). TPS implants demonstrated less favorable long-term stability, even with grafting (27). Smooth (turned) surfaces were rarely evaluated in regenerative contexts, limiting conclusions. Khoury & Buchmann reported substantial bone gain (3.2 mm) on rough implants with autografts (20), while Roos-Jansåker et al. found stable bone gain (1.1–1.6 mm) in predominantly machined implants (29, 30).

### 3.5 Subgroup analyses

#### 3.5.1 Turned surfaces

Showed moderate long-term stability, but implant loss remained high when treated non-reconstructively (23). Reconstructive data were limited but suggested stable outcomes (29, 30).

#### 3.5.2 Modified (rough) surfaces

Non-reconstructive approaches resulted in high recurrence and implant loss (15, 16). Reconstructive approaches improved outcomes, with SLA surfaces outperforming TPS (26, 27).

#### 3.5.3 Mixed surfaces

Outcomes were heterogeneous. Laser-assisted non-reconstructive therapy demonstrated short-term benefits Deppe et al. (18), but Jemt & Eriksson reported long-term bone loss regardless of surface type (19). Reconstructive treatments showed better results with natural bone mineral combined with a collagen membrane (NBM + CM) compared to nanocrystalline hydroxyapatite (NHA) (31), but surface-specific differences remained underreported. Aghazadeh et al. reported improved outcomes with xenograft (BDX) usage (32).

The detailed study characteristics and outcomes are presented in Tables 1a,b.

### 3.6 Risk of bias

The risk of bias assessment for the 17 included studies highlights several concerns across different domains (Tables 3a and b).

**Table 3a:** Risk of bias assessment for RCTs: RoB 2 risk of bias assessment.

Study	Bias arising from the randomization process	Bias due to deviations from intended interventions	Bias due to missing outcome data	Bias in measurement of the outcome	Bias in selection of the reported result	Overall bias
Schwarz 2009 (31)	Low	Low	Some concerns	Low	Low	Some concerns

**Table 3b:** Risk of bias assessment for non- RCTs: ROBINS-i risk of bias assessment.

Study	Bias due to confounding	Bias in selection of participants into the study	Bias in classification of interventions	Bias due to deviations from intended interventions	Bias due to missing data	Bias in measurement of outcomes	Bias in selection of reported results	Overall bias
Aghazadeh 2022 (32)	Moderate	Serious	Low	Moderate	Serious	Moderate	Low	Serious
Carcuac 2020 (15)	Moderate	Serious	Low	Moderate	High	Low	Low	Serious
Deppe 2007 (18)	Moderate	Moderate	Low	Moderate	Serious	Moderate	Low	Serious
Jemt et al. 2021 (19)	Moderate	Serious	Low	Moderate	Serious	Moderate	Low	Serious
Khoury 2001 (20)	Serious	Low	Moderate	Moderate	Low	Moderate	Low	Moderate
La Monaca 2018 (21)	Moderate	Moderate	Low	Moderate	Serious	Low	Low	Moderate
La Monaca 2024 (22)	Serious	Serious	Low	Moderate	Serious	Moderate	Low	Serious
Leonhardt 2003 (23)	Moderate	Low	Low	Moderate	Low	Moderate	Low	Moderate
Mercado 2018 (24)	Moderate	Low	Low	Moderate	Low	Low	Low	Moderate
Noelken 2023 (25)	Moderate	Moderate	Low	Moderate	Serious	Moderate	Low	Moderate
Roccuzzo 2017 (26)	Moderate	Moderate	Low	Moderate	Moderate	Low	Low	Moderate
Roccuzzo 2020 (27)	Moderate	Serious	Low	Moderate	Serious	Low	Low	Serious
Roccuzzo 2021 (28)	Moderate	Serious	Low	Moderate	Serious	Moderate	Low	Serious
Romandini 2024 (16)	Moderate	Serious	Low	Moderate	Serious	Moderate	Low	Serious
Roos-Jansaker 2011 (29)	Moderate	Serious	Low	Moderate	Serious	Low	Low	Serious
Roos-Jansaker 2014 (30)	Moderate	Serious	Low	Moderate	Serious	Low	Low	Serious

The RCT (Schwarz 2009) had some concerns (31).

Among the non-randomized studies, serious risk of bias was frequently noted in selection of participants e.g., Aghazadeh, Carcuac, and La Monaca (15, 17, 22) and missing data e.g., Roccuzzo, Romandini, Roos-Jansaker (16, 27, 30). However, intervention classification and reporting of results were generally at low risk across studies. Confounding and deviations from interventions were rated as moderate risk in most cases.

Ten studies were judged to have a serious overall bias, primarily due to participant selection and missing data. The remaining studies had a moderate overall bias, with issues mainly related to confounding and missing data.

Certainty of evidence for the main outcomes was assessed using the GRADE approach and is presented in Supplementary Table S1. Overall, the certainty was judged to be very low to low across all outcomes, primarily due to serious risk of bias, high heterogeneity of diagnostic criteria and outcome definitions, small sample sizes, and imprecision of effect estimates.

## 4 Discussion

This systematic review aimed to evaluate the impact of implant surface characteristics on the long-term outcomes of surgical treatment of peri-implantitis. The main findings indicate that modified (rough) surfaces are consistently associated with higher recurrence and implant loss compared with turned (machined) surfaces. Within rough surfaces, SLA implants achieved more favorable outcomes than TPS, particularly in reconstructive contexts (15, 16, 26–28). In contrast, smooth implants demonstrated comparatively lower recurrence (23). These results underscore that implant surface topography can be a determinant of surgical treatment prognosis.

The effectiveness of peri-implantitis surgery is influenced by both treatment modality and implant surface. For non-regenerative procedures, modified surfaces were repeatedly associated with worse outcomes: Carcuac et al. reported a 44% recurrence rate with rough implants (15), while Romandini et al. identified modified surfaces such as TiUnite and SLA as predictors of implant loss (16). In contrast, turned surfaces showed more stable disease suppression (23).

For regenerative approaches, implant surface also played a key role. SLA implants demonstrated favorable long-term bone gain and PD reduction (26–28), whereas TPS implants performed poorly even when grafting was applied (27). Although smooth implants were less frequently studied in regenerative contexts, available evidence suggests they may perform adequately when combined with supportive therapy (29, 30).

Bone regeneration outcomes differed substantially according to surface characteristics. Greater bone fill was generally reported around rough surfaces when grafting materials were used. Khoury and Buchmann observed a 2.4 mm gain at 12 months using autogenous grafts on rough implants (20). Roccuzzo et al. reported significant defect reduction with xenografts, particularly in SLA implants, while TPS implants showed limited stability (26, 27). Comparable results with alloplastic materials were also noted (33, 34). However, bone regeneration around smooth surfaces was less favorable: Roos-Jansåker et al. reported limited improvement with alloplastic grafts (30). Thus, while rough implants may predispose to recurrence, they also appear to support more pronounced bone regeneration after reconstructive procedures.

This paradox may be explained by surface-related biology. Rough surfaces are harder to decontaminate and accumulate more plaque (35, 36), yet they may stabilize the coagulum and promote defect fill (37). Accordingly, radiographic bone gain does not necessarily correspond to re-osseointegration, as several animal studies identified connective tissue interposition rather than true reattachment (38–40).

The role of membranes in guided bone regeneration (GBR) has also been linked to implant surfaces. Khoury et al. showed greater bone gain with non-resorbable membranes around rough implants (20), while Deppe et al. observed comparable results with resorbable membranes (18). These data suggest that both membrane type and surface roughness influence regenerative outcomes. Furthermore, clinical studies and experimental models in dogs indicate that rough surfaces generally achieve greater defect fill than smooth surfaces under GBR conditions (37).

Surface characteristics may also impact soft tissue attachment. Excessively smooth surfaces can impair mucosal adhesion, as Quirynen et al. observed attachment loss on polished abutments compared with stable CAL around commercially available surfaces (41). Other studies support that maintaining a certain degree of roughness enhances soft tissue sealing (42). These findings provide a biological explanation for the improved clinical outcomes of rough implants after GBR, despite their higher susceptibility to recurrence.

Interpretation of the evidence is complicated by considerable heterogeneity. Defect morphology influences outcomes, with narrower defects showing better results (17, 43), yet most studies failed to provide detailed descriptions, limiting cross-study comparisons. Moreover, peri-implantitis definitions varied widely: Roccuzzo et al. required ≥6 mm PD and bone loss exceeding three implant threads (26), while Mercado et al. used ≥4 mm PD and ≥20% radiographic bone loss (24). Measurement variability further complicates interpretation (44). Such inconsistencies directly affect assessment of surface-related outcomes and hinder robust comparisons across studies.

This review is limited by the substantial heterogeneity among the included studies, particularly in peri-implantitis diagnostic criteria, defect morphology, surgical techniques, and outcome measures. Most studies were small in size, lacked standardized definitions, and many were judged to have a serious overall risk of bias, especially in participant selection and missing data. Confounding variables were insufficiently controlled, further reducing certainty. Furthermore, the restriction to English-language studies may have introduced language bias, potentially leading to omission of relevant non-English publications. Applying the GRADE framework, the certainty of the available evidence was rated as very low to low for all main outcomes, reflecting methodological shortcomings and heterogeneity among the included studies. These limitations restrict the generalizability of the findings and reinforce the need for well-designed, adequately powered randomized controlled trials with standardized definitions and longer follow-up.

## 5 Conclusion

The effectiveness of peri-implantitis surgery is influenced by implant surface characteristics and treatment modality. Modified surfaces are generally more prone to recurrence and implant loss, with SLA implants performing better than TPS, while turned surfaces appear less susceptible but remain insufficiently studied in regenerative contexts. Reconstructive approaches combined with supportive care consistently provide the most favorable outcomes. Given the very low to low certainty of the evidence with heterogenous results, current findings should be interpreted with caution, and well-designed long-term randomized trials with standardized definitions and consistent surface classifications are urgently needed. Future trials should adopt standardized outcome definitions (e.g., PD thresholds, BOP, radiographic bone loss criteria) to allow comparability across studies. Research should focus on RCTs directly comparing surface types, long-term follow-up, and adjustment for confounding factors such as defect morphology and maintenance compliance. Addressing these gaps will clarify the role of implant surface modifications.

### 5.1 Clinical implications

When planning peri-implantitis surgery, implant surface characteristics should be taken into account, but they must not be considered in isolation. Evidence indicates that reconstructive approaches yield more reliable outcomes than non-reconstructive ones, particularly for rough implants, with SLA surfaces performing more favorably than TPS. Turned (machined) surfaces appear less prone to recurrence, although data on regenerative protocols remain scarce. These observations suggest that implant surface may influence prognosis, yet it represents only one part of a complex clinical picture.

Patient-related risk factors (such as smoking, systemic conditions, low compliance and/or adherence to supportive care) exert a profound effect on long-term success and may outweigh surface-related differences. Surgical decision-making should therefore be individualized, integrating implant surface type, defect morphology, patient risk profile, and anticipated compliance. The use of biomaterials and barrier membranes may enhance regenerative outcomes around rough implants, but clinicians should be cautious, as radiographic bone gain does not necessarily reflect true re-osseointegration, and complete defect resolution is rarely achievable.

Nevertheless, these clinical implications must be interpreted with caution. The available evidence is heterogeneous, often based on small studies with differing peri-implantitis definitions, inconsistent outcome measures, and a serious overall risk of bias. The evidence was rated as very low to low for all main outcomes. This means that while current data can guide clinical choices, they cannot provide definitive recommendations.

## Data Availability

The original contributions presented in the study are included in the article/Supplementary Material, further inquiries can be directed to the corresponding author.

## Appendix

**Appendix 1:** Excluded studies.

Authors and date	Study title	Reason for exclusion
Afrashtehfar et al. 2024 (45)	Guided bone regeneration improves defect fill and reconstructive outcomes in 3-wall peri-implantitis defects	Implant type not taken into consideration
Astolfi et al. 2021 (46)	Influence of removing or leaving the prosthesis after regenerative surgery in peri-implant defects: retrospective study: 32 clinical cases with 2–8 years of follow-up	Outcomes reported not correlated to implant type
Behneke et al. 2000 (47)	Treatment of peri-implantitis defects with autogenous bone grafts: six-month to 3-year results of a prospective study in 17 patients	Implant type not taken into consideration
Berglundh 2018 (48)	Long- term outcome of surgical treatment of periimplantitis. A 2–11-year retrospective study	Minimum study duration less than 3 years
Bianchini et al. 2020 (49)	Implantoplasty enhancing peri-implant bone stability over a 3-year follow-up: a case series	Implant type not taken into consideration
Bianchini et al. (2024) (50)	Clinical and radiographic outcomes of resective surgery with adjunctive implantoplasty over a 6- to 11-year follow-up: a case series	Implants treated with implantoplasty, which creates a modified surface texture differing from the original implant surface
Carcuac et al. 2016 (51)	Adjunctive systemic and local antimicrobial therapy in the surgical treatment of peri-implantitis: a randomized controlled clinical trial	Study duration less than 3 years
Chiang et al. 2024 (52)	Operating microscope-assisted reconstructive strategy for peri-implantitis: A case series report	Implant type not taken into consideration
Cortellini et al. 2021 (53)	Papilla preservation and minimally invasive surgery for the treatment of peri-implant osseous defects. Clinical and radiographic outcomes of a 5-year retrospective study	Implant type not taken into consideration
Froum et al. 2012 (54)	Successful management of peri-implantitis with a regenerative approach: a consecutive series of 51 treated implants with 3- to 7.5-year follow-up	Outcomes mentioned not correlated to implant surfaces
Froum et al. 2015 (55)	A regenerative approach to the successful treatment of peri-implantitis: a consecutive series of 170 implants in 100 patients with 2- to 10-year follow-up	Implant type not taken into consideration
Khayat et al. 2024 (56)	Bone regeneration following implantoplasty: a retrospective cohort study with long-term radiographic assessment	Implant type not taken into consideration
Lombardo et al. 2019 (57)	Successful management of peri-implantitis around short and ultrashort single-crown implants: a case series with a 3-year follow-up	Implant type not taken into consideration
Monje et al. 2022 (58)	Principles of combined surgical therapy for the management of peri-implantitis	Incorrect study design
Parma-Benfenati et al. 2020 (59)	Long-term outcome of surgical regenerative treatment of peri-implantitis: a 2- to 21-year retrospective evaluation	Study duration less than 3 years (varied for 2–21 years)
Renvert et al. 2012 (60)	Surgical therapy for the control of peri-implantitis	Incorrect study design
Renvert et al. 2024 (61)	The efficacy of reconstructive therapy in the surgical management of peri-implantitis: A 3-year follow-up of a randomized clinical trial	Implant type not taken into consideration
Sarmiento et al. 2018 (62)	Surgical alternatives for treating peri-implantitis	Implant type not taken into consideration
Schwarz et al. 2015 (63)	Reentry after combined surgical resective and regenerative therapy of advanced peri-implantitis: a retrospective analysis of five cases	Implant type not taken into consideration
Schwarz et al. 2014 (64)	Combined surgical therapy of advanced peri-implantitis lesions with concomitant soft tissue volume augmentation. A case series	Study duration less than 3 years
Schwarz et al. 2013 (65)	Four-year follow-up of combined surgical therapy of advanced peri-implantitis evaluating two methods of surface decontamination	Implants treated with implantoplasty, which creates a modified surface texture differing from the original implant surface
Schwarz et al. 2017 (66)	Combined surgical therapy of advanced peri-implantitis evaluating two methods of surface decontamination: a 7-year follow-up observation	Implants treated with implantoplasty, which creates a modified surface texture differing from the original implant surface
Wang et al. 2021 (67)	Laser-assisted regenerative surgical therapy for peri-implantitis: A randomized controlled clinical trial	Study duration less than 3 years

**Appendix 2:** Full search strategies.

Database	Search Strategy
PubMed (MEDLINE via PubMed)	(Periimplantitis OR peri-implantitis OR peri implantitis OR periimplant OR peri-implant OR peri implant) AND (treatment outcome OR therapy OR surgical treatment OR regenerative OR regeneration OR tissue regeneration OR reconstructive surgery OR bone graft OR bone substitute OR membranes OR surgical flap OR open flap debridement OR resective OR implantoplasty OR surface decontamination) AND (surface characteristics OR surface roughness OR material characteristics OR titanium surface OR implant types OR implant surfaces OR surface topography OR surface analysis) AND (implant survival OR bone loss OR recurrence OR retreatment OR radiographic stability OR long-term OR 3 years OR follow-up)
Embase	(‘periimplantitis’/exp OR periimplantitis OR ‘peri-implantitis’ OR ‘peri implantitis’ OR periimplant OR ‘peri-implant’ OR ‘peri implant’) AND (‘treatment outcome'/exp OR therapy OR ’surgical treatment'/exp OR ‘regenerative therapy'/exp OR regeneration OR ‘tissue regeneration’ OR ‘reconstructive surgery'/exp OR ‘bone graft'/exp OR ‘bone substitute'/exp OR membranes OR ‘surgical flap’ OR ‘open flap debridement’ OR resective OR implantoplasty OR ‘surface decontamination’) AND (‘surface property'/exp OR ‘surface roughness'/exp OR ‘material property'/exp OR ‘titanium surface’ OR ‘implant type'/exp OR ‘implant surface'/exp OR ‘surface topography'/exp OR ‘surface analysis’) AND (‘dental implant survival'/exp OR ‘bone loss'/exp OR recurrence OR retreatment OR ‘radiographic stability’ OR ‘long term’ OR ‘3 years’ OR ‘follow-up’)
Cochrane library	(periimplantitis OR “peri-implantitis” OR “peri implantitis” OR periimplant OR “peri-implant” OR “peri implant”) AND (“treatment outcome” OR therapy OR “surgical treatment” OR regenerative OR regeneration OR “tissue regeneration” OR “reconstructive surgery” OR “bone graft” OR “bone substitute” OR membranes OR “surgical flap” OR “open flap debridement” OR resective OR implantoplasty OR “surface decontamination”) AND (“surface characteristics” OR “surface roughness” OR “material characteristics” OR “titanium surface” OR “implant types” OR “implant surfaces” OR “surface topography” OR “surface analysis”) AND (“implant survival” OR “bone loss” OR recurrence OR retreatment OR “radiographic stability” OR “long term” OR “3 years” OR “follow-up”)
