# Supplementary material for: Impact of implant surface modifications on long-term outcome of surgical peri-implantitis treatment: a systematic review
Source: Front Dent Med. 2025 Sep 24;6:1661369. doi: 10.3389/fdmed.2025.1661369 (PMC12504269; doi:10.3389/fdmed.2025.1661369)
Supplement: Supplementary file 1 [file Table1.docx]

# Supplementary Table S1. GRADE assessment of certainty of evidence for main outcomes

| Outcome | Number of studies | Risk of bias | Inconsistency | Indirectness | Imprecision | Publication bias | Overall certainty of evidence |
| --- | --- | --- | --- | --- | --- | --- | --- |
| Recurrence of peri-implantitis after surgery | 17 | Serious | Serious (heterogeneous criteria, defect morphology) | Not serious | Serious (small samples, wide CIs) | Possible | Very low |
| Implant loss (any cause) | 15 | Serious | Moderate (heterogeneity present but direction consistent) | Not serious | Serious (low event rates, underpowered) | Possible | Low |
| Disease resolution (PD reduction + no BOP/SOP) | 16 | Serious | Serious (non-standardized definitions, varied outcomes) | Not serious | Serious | Likely | Very low |
| Radiographic bone gain/loss | 14 | Serious | Serious (different radiographic methods, thresholds) | Not serious | Serious (imprecise estimates) | Likely | Very low |
| Mean PD reduction (mm) | 16 | Serious | Moderate (direction consistent, magnitude varied) | Not serious | Serious (small samples, wide SDs) | Possible | Low |

Legend: Certainty ratings follow GRADE (High, Moderate, Low, Very low). Downgrading factors considered: risk of bias (ROBINS-I and RoB2), inconsistency (heterogeneity of criteria), indirectness (population and interventions relevant), imprecision (small samples, wide confidence intervals), and potential publication bias.
